# Strength and microstructural properties of silt soil cured by lime-activated fly ash-GGBS under different curing temperatures

**DOI:** 10.1038/s41598-024-57741-4

**Published:** 2024-03-23

**Authors:** Shunmei Gong, Songbao Feng, Shiquan Wang, Lemei Yu, Yuanyuan Chen, Qiang Xu

**Affiliations:** 1 National Engineering Research Center of Coal Mine Water Hazard Controlling, School of Resources and Civil Engineering, Suzhou University, Suzhou, 234000 China; 2 Jiangsu Weixin Engineering Consulting Co., Ltd., Nanjing, 210014 Jiangsu China

**Keywords:** Curing temperature, Fly ash-GGBS, Unconfined compressive strength, Hydration reaction, Pozzolanic reaction, Environmental sciences, Solid Earth sciences, Energy science and technology, Engineering, Materials science

## Abstract

To reveal the mechanism of the influence of the curing temperature on the strength of lime activated fly ash-GGBS cured silt soil, the curing of dredged silt was carried out by using fly ash and GGBS as the curing agent and lime as the activator. Unconfined compressive strength (UCS) experiments were carried out, and the micro-analysis of the cured silt was carried out by experimental methods including scanning electron microscope (SEM) tests, X-ray diffraction (XRD), etc. to reveal the mechanism of the curing temperature on the dredged silt. According to the test results, the hydration reaction and pozzolanic reaction between lime-fly ash-GGBS and silt soil were promoted with the increase of the curing temperature. when the curing temperature of the sample reached 40 ℃, a large amount of gel products such as hydrated calcium aluminate (C–A–H) and hydrated calcium silicate (C–S–H) were generated, which enhanced the bonding force between soil particles and filled up the inter-particle pore space, thereby improving the UCS of the sample. The results of SEM confirmed that C–A–H and C–S–H were the main substances for the construction of cured silt skeleton. C–S–H and C–A–H were detected by XRD. The results of the study fill the gap in the effect of curing temperature on the direction of lime-activated fly ash-GGBS cured silt soil.

## Introduction

A large amount of dredged silt (DS) will be generated in water environment governance and urban river dredging projects^1,2^. Most of the dredged silt is in the fluid-plastic state, with high water content and dismal mechanical properties, making it unable to be effectively utilized in engineering construction. Cement, as the most commonly used high-efficiency curing agent, exhibits many problems in the production process such as huge CO_2_ emissions and high energy consumption, resulting in serious pollution to the environment^3^. In recent years, the concepts of green environmental protection, as well as energy saving and emission reduction in the treatment of waste sludge have gradually been highly valued by scholars at home and abroad. The energy consumption of cement-cured sludge is 160% of that of alkaline-activated cement-cured sludge^4^. It can be seen that alkaline activators can interact with some active substances in industrial by-products to generate hydration products with gelation effect, thereby reducing energy consumption. Therefore, the green and low-carbon new curing agent can replace or partially replace the traditional curing agent with high energy consumption, high emission and high pollution, which has become a research hotspot strengthening treatment and reuse of waste silt at the current stage^5^.

Domestic and foreign scholars have attempted to develop new curing agents by using industrial by-products as the main raw materials, which have achieved abundant research results. Industrial solid wastes such as carbide slag, fly ash, GGBS and acetylene sludge can be used as curing agents for dredging sludge, which can partially replace the cement curing agent with high emission, high pollution and high energy consumption^6,7^. According to the current market price, the price of GGBS and fly ash is 30% and 50% of the price of cement, respectively. Literature^8^ conducted UCS experiments and found that the addition of fly ash to cured silt could effectively improve the strength of cured silt. Literature^9^ found that elevating curing temperature could significantly promote the hydration rate of cement-cured silt and generate cementitious products with improved cured strength. In terms of traditional curing agents, Bell^10^ studied the properties and mechanisms of lime-cured soil, with the increase of the curing temperature, the strength of lime-cured soil increases significantly, and when the curing temperature is higher than 30℃, the changing rate of its strength increases significantly. Al-Mukhtar et al.^11,12^ studied the mechanism and properties of the effect of lime modified expansive soils, respectively setting up the 20 ℃ and 50 ℃ curing conditions, and the results of the study showed that the increase of the curing temperature accelerated the pozzolanic reaction between the substances and promoted the rapid increase of the strength of the improved soil. Liu et al.^13^ studied the mechanism of the influence of the curing temperature, the age of curing and the lime admixture on the UCS of the cured soft soil, and the study found that the strength of the cured soil increased with the increase of the curing temperature and that there existed a certain index between them. The study found that the strength of cured soil increases with the increase of curing temperature, and there is an exponential relationship between the two. The hydration reaction rate of cement cured soil is closely related to the change of curing temperature. Zhang et al.^14^ investigated the influence law of the curing temperature on the strength of cement-cured silt, with the increase of curing temperature, the rate of pozzolanic reaction and chemical reaction of cement-cured silt was accelerated, thus increasing the strength of cement-cured silt. Considering the effect of temperature, a new design method of cement-cured silt ratio was proposed. Chen et al.^15^ found a certain exponential relationship between the strength and deformation modulus of cemented silt at different curing temperatures by investigating the law of curing temperature on the strength of cemented silt. In terms of new curing agents, Phetchuay^16^ investigated the mechanism of the effect of calcium carbide GGBS and fly ash on the strength of silt soil. The results showed that when the curing temperature increased from room temperature of 25–40 ℃, the UCS of the cured soil increased significantly. Consoli et al.^17^ used GGBS and rice husk ash as refiners of sandy soil, and found that the curing temperature could significantly increase the pozzolanic reaction between GGBS and rice husk ash. Moreover, the UCS of the amended sandy soil at 40 ℃ for 7 days was close to that of the amended sandy soil maintained at 23 ℃ for 28 days. He et al.^18^ studied the geotechnical properties of GGBS and lime curing of waste sludge, and found that the cured sludge exhibited the highest strength at 40 ℃. Zhang et al.^19^ found that the early and long-term strengths of cement-cured sludge increased correspondingly with the increase of curing temperature, which also enhanced the rate of pozzolanic reaction and chemical reaction.

In view of this, the strength development of silt soil cured with new curing agents such as cement, lime, GGBS, and industrial waste are all related to the temperature. Through numerous experimental studies, the author found that GGBS and fly ash can be used as a new type of curing agent for silt, with more significant curing effect. However, the effect of the curing temperature on the lime-activated fly ash-GGBS cured silt soil remains unclear. In actual engineering, the ambient temperature is higher than 20 ℃ for at least more than half a year, and the ambient temperature in summer can reach up to 40 ℃. There are fewer researches on the law of the mechanical behavior of the cured soil induced by the high temperature often encountered in the actual engineering practice, especially in the consideration of even fewer studies on the strength and microstructure of silt soil cured by lime-activated fly ash-GGBS under different curing temperatures. Therefore, it is of great significance to systematically study the influence mechanism of curing temperature on lime-activated fly ash-GGBS cured silt. In this study, the lime-GGBS-fly ash cured waste silt was taken as the research object. UCS of the cured silt under the conditions of different curing ages and curing temperatures, as well as tests such as SEM, XRD, etc. were carried out to study the influence of different curing temperatures on the strength of silt soils, so as to provide research basis for resource utilization of fly ash and GGBS.

## Materials and methods

### Materials

**Figure 1:**
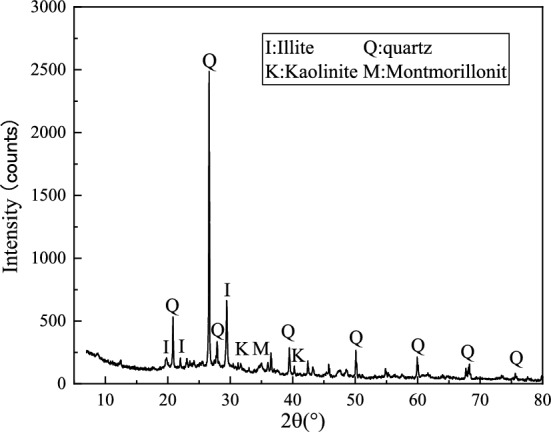
XRD pattern of the silt.

Waste silt from the dredging site of silt removal in a designated section of the river in Suzhou City was selected as the experimental soil. The original silt was gray-black in color, with a natural moisture content of 60% and a pore ratio of 1.19. The waste silt was subjected to chemical pretreatment after retrieval, then dried and crushed into powder, and finally passed through a 1 mm geotechnical sieve for backup. According to the specifications^20–23^, various indicators of silt were standardized and measured, as shown in Table 1. The experimental silt mainly contains illite, quartz, kaolinite, and montmorillonite; lime mainly contains calcium oxide and magnesium oxide; fly ash mainly contains quartz and mullite; and GGBS mainly contains calcium-aluminum xanthite feldspar, dicalcium silicate, and tricalcium aluminate. The mineral composition of silt soil measured by XRD is shown in Fig. 1. Lime, fly ash and GGBS were in powder form and the chemical composition was determined by XRD and the measurements are shown in Table 2.

**Table 1:** Characterization of experimental silt.

Parameters	Values	Parameters	Values
Liquid limit (%)	50.0	Sand fraction (> 0.075 mm) (%)	10.95
Specific gravity	2.69	Silt fraction (0.005 mm < % < 0.075 mm) (%)	63.25
Plastic index (%)	25.02	Clay fraction (< 0.005 mm) (%)	14.85
Plastic limit (%)	23.9	Organic matter content (%)	1.55

**Table 2:** Chemical composition of raw materials.

Raw materials	CaO (%)	SiO_2_ (%)	Al_2_O_3_ (%)	Fe_2_O_3_ (%)	MgO (%)	Others (%)
Lime	85.76	3.38	2.30	0.40	6.02	2.14
Fly ash	2.51	53.38	26.96	3.30	4.34	9.51
GGBS	41.80	33.20	12.30	0.70	6.20	5.80

### Test scheme

**Figure 2:**
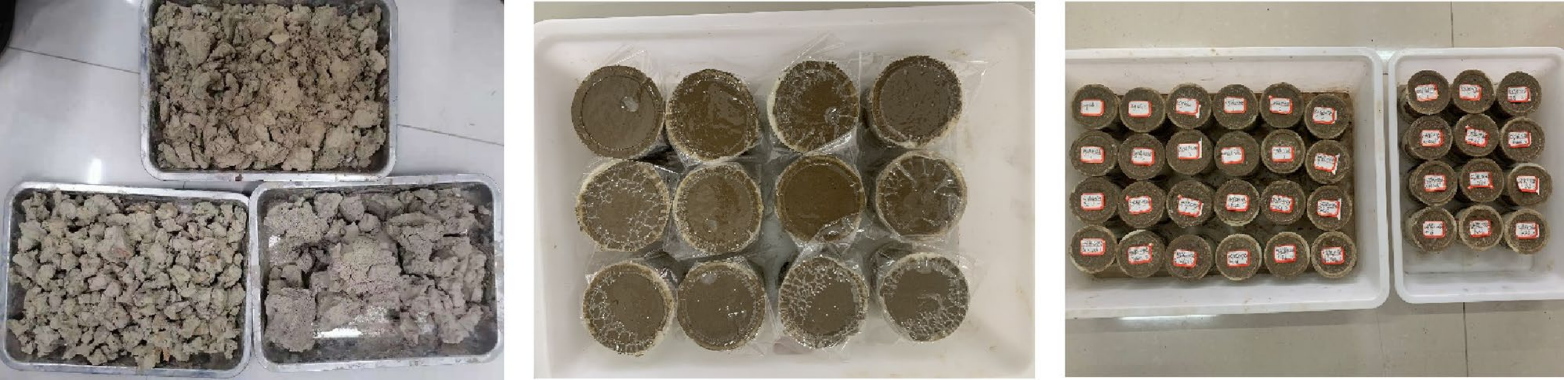
Solidified silt sample.

During the experiment, the spare silt soil was taken out and activated with water according to the set water content of 60%. In previous studies, the addition of 10% curing agent has been able to meet the cured strength objectives for soft soil. Lime, as the alkaline stimulant of fly ash and GGBS, provides abundant hydroxide ions and accounts for 20% of the total curing agent, while fly ash and GGBS, as the main curing agent, account for 80% of the total curing agent. The addition of 20% lime is just enough to fully stimulate the chemical reaction between fly ash and GGBS. At the same time, it saves costs and makes efficient use of industrial waste, with a view to realizing the goal of green development. Thus, the total amount of curing agent was 10%, and lime accounted for 20%. The remaining 80% was added according to different proportions of fly ash and GGBS. A control group with pure cement as curing agent was established, with a content of 10%. The lime, fly ash and GGBS were dried at 60 °C and sieved by 1 mm sieve, then mixed together with sludge in a mortar mixer for 10 min until uniform. The sample was prepared after sealing and standing for 12 h. Three parallel samples were prepared for each ratio to ensure the repeatability, accuracy and fault tolerance of the experiment. By referring to the literature^24,25^, a specific test program was developed as shown in Table 3. Fly ash is represented by the symbol FA, and slag is represented by the symbol GGBS. In the table, FA:GGBS that the ratio of fly ash and slag is 10:0, which is abbreviated as F10G0 below, and the meaning of other symbols is by analogy. Part of the silt sample is shown in Fig. 2 below.

**Table 3:** Test program.

Initial water content	Cement content/%	Lime content/%	FA:GGBS	Symbol	Curing temperature/^o^C	Curing age/days
The liquid limit is 50%. The initial water content is 60%, which is 1.2 times the liquid limit		20	10: 0	F10G0	20, 30, 40	3, 7, 28, 90
			8:2	F8G2		
			6:4	F6G4		
			4:6	F4G6		
			2:8	F2G8		
			0:10	F0G10		
	10			ce	20	3, 7, 28, 90

Notes: F10G0 indicates that the ratio of fly ash to GGBS is 10:0, abbreviated as FA:GGBS = 10:0. The meanings of other symbols are similar.

## Results and analysis

### UCS

#### Effect of 20 °C curing temperature on the UCS of silt soil

**Figure 3:**
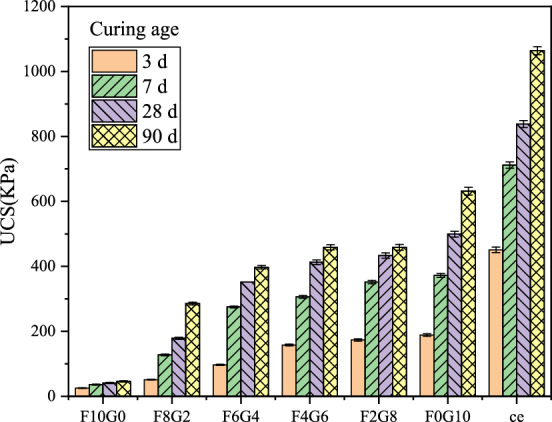
The influence of different curing agents on the strength of silt soil under normal temperature curing at 20 °C.

When the curing temperature of the sample was set to 20 °C, the ratio of fly ash and GGBS was set to 10:0, 8:2, 6:4, 4:6, 2:8 and 0:10, respectively. Under the combined action of the three curing agents, the strength of silt soil showed an upward trend as the proportion of GGBS increased. At the same time, the strength of silt soil increased with the increase of age. When the content of cement curing agent was 10%, the UCS of the original sludge solidified body was 450, 711, 838, and 1063 kPa, respectively at 3, 7, 28 and 90 days of curing, respectively. Figure 3 shows the influence law of UCS of silt soil under the curing condition of 20 °C at normal temperature. In the figure, ce represents the cement curing agent.

#### Effect of 30 °C curing temperature on the UCS of silt soil

**Figure 4:**
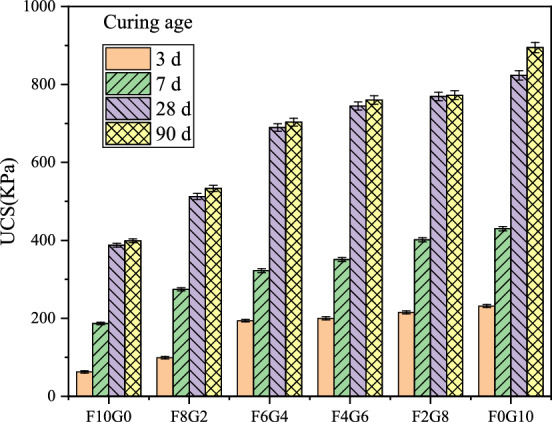
The influence of different curing agents on the strength of silt soil under temperature curing at 30 °C.

When the curing temperature of the sample was set to 30 °C, the experimental method was the same as above. Under the combined action of the three curing agents, the strength of the silt soil still shows an upward trend with the increase of the proportion of GGBS. At the same time, the strength of silt soil increased with the increase of age. Notably, the 90-day strength of the F0G10 sample was 1.1 times that of the original sludge solidified body with 10% cement as curing agent when cured to 28 days under the condition of a curing temperature of 30 °C, indicating that the alkali-activated waste GGBS could partially replace the cement curing agent as the curing temperature increased. The influence law of UCS of silt soil is shown in Fig. 4.

#### Effect of 40 °C curing temperature on the UCS of silt soil

**Figure 5:**
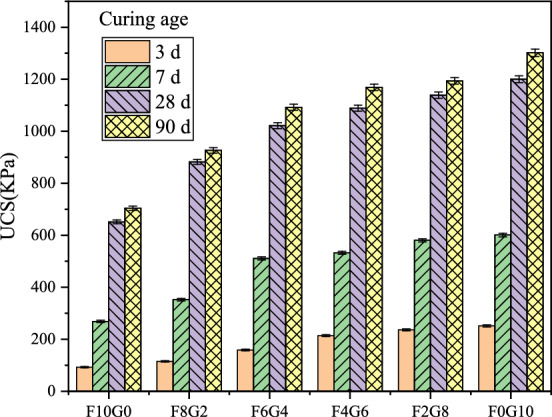
The influence of different curing agents on the strength of silt soil under temperature curing at 40 °C.

When the curing temperature of the sample was set to 40 °C, the experimental method was the same as above. Under the combined action of the three curing agents, the strength of the silt soil shows an upward trend again with the increase of the proportion of GGBS. It is worth noting that when the curing temperature T = 40 °C, the 28-day strength of F8G2, F6G4, F4G6, F2G8 and F0G10 was 882, 1021, 1088, 1139, and 1200 kPa, respectively, which has exceeded the UCS of the original sludge solidified body with 10% cement as the curing agent when cured to 28 days. The 90-day strength of F6G4, F4G6, F2G8 and F0G10 was 1092, 1169, 1189, and 1300 kPa, which has exceeded the UCS of the original sludge solidified body when cured to 90 days. It is further indicated that the UCS of the silt soil sample increases as the curing temperature rises. It is confirmed that with the increase of temperature, the waste fly ash and GGBS can partially replace the expensive cement and save the curing cost. The influence law of UCS of silt soil is shown in Fig. 5.

#### UCS growth rate analysis

To further analyze the effect of curing temperature on the strength of lime-activated fly ash-GGBS cured silt soil, the strength growth rate ($\theta_{T}$,%) is defined as:

1 $$\theta_{T} { = }\frac{{\beta_{x} - \beta_{{20^{ \circ } C}} }}{{\beta_{{20^{ \circ } C}} }} \times 100{\text{\% }}\;\;\;\left( {x\; = \;30^{ \circ } C,\;40^{ \circ } C} \right)$$

where $\theta_{T}$ in the formula indicates that the strength growth rate, which can reflect the influence of curing temperature on the strength of silt samples; $\beta_{x}$ indicates the strength of the silt samples when the curing temperature is 30 ℃ or 40 ℃;$\beta_{{20^{ \circ } C}}$ indicates the strength of the silt samples when the curing temperature is 20 ℃.

**Figure 6:**
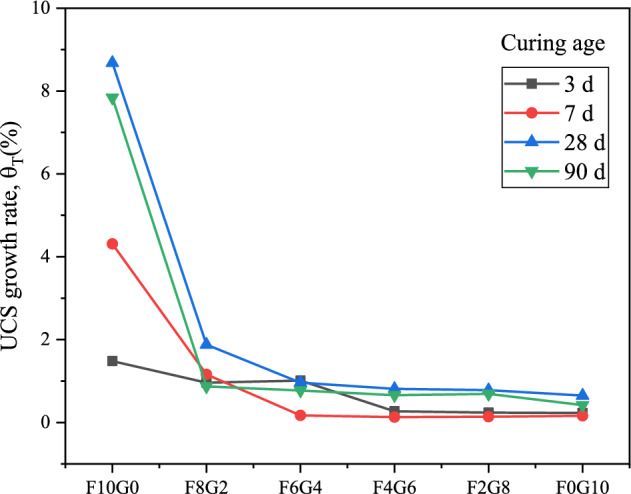
UCS growth rate of silt samples when sample curing temperature is increased from 20 to 30 °C.

**Figure 7:**
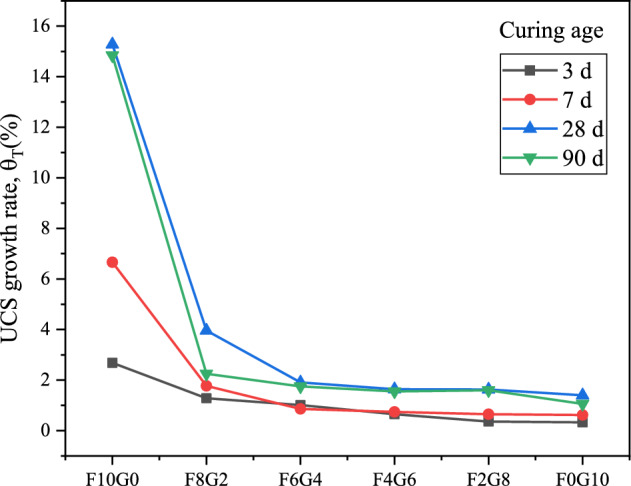
UCS growth rate of silt samples when sample curing temperature is increased from 20 to 40 °C.

As can be seen from Figs. 6 and 7, the strength of the samples showed a growth trend with the increase of the curing temperature. The 28-day strength growth rate of sample F10G0 reached 8.68 times when the sample curing temperature was increased from 20 to 30 °C; the 28-day strength growth rate of sample F10G0 reached 15.28 times when the sample curing temperature was increased from 20 to 40 °C. The analysis of the strength growth rate of silt samples indicates that the effect of lime stimulating fly ash is more significant with the increase of the curing temperature. As the proportion of GGBS increased, the active CaO, SiO_2_ and Al_2_O_3_ in the GGBS participated in the hydration reaction and pozzolanic reaction, and accelerated the rate of chemical reaction between the mixed substances with the increase of the curing temperature. The strength growth rate of the samples at 28 days and 90 days is greater than that of the early strength, which further indicates that the curing temperature is more effective in increasing the strength of the silt cured body in the middle and late stages.

### SEM analysis

**Figure 8:**
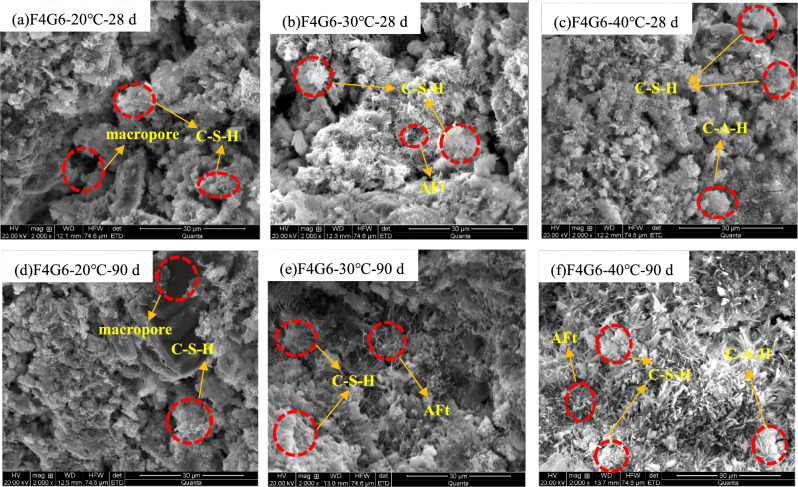
SEM images of sample F4G6 at different curing temperatures at 28 days and 90 days. Notes: F4G6-20℃-28 d means SEM micrographs of sample F4G6 at a curing temperature of 20 ℃ for 28 days; F4G6-20℃-90 d means SEM micrographs of sample F4G6 at a curing temperature of 20 ℃ for 90 days. The meanings of other symbols are similar.

Under the conditions of curing temperature of 20 °C, 30 °C and 40 °C, the representative SEM photos of the sample cured for 28 days are shown in Fig. 8a–c (taking sample F4G6 as an example). When the curing temperature T = 20 °C, irregular spherical C–S–H could be observed when the finer particles were filled in the pore. After 2000 times magnification by SEM, the particle boundary was blurred. When the curing temperature increased from 20 to 30 °C, the number of needle-like ettringite sharply increased, and the particles were intertwined by hydration products to form a relatively dense network structure, and foil-like C–S–H could also be observed, indicating that the strength was improved to a certain extent. Under the condition that the curing temperature reached 40 °C, the morphology of ettringite in the sample changed, mostly in a short rod-like form, and the observed ettringite crystals decreased somewhat, which may be due to the fact that the solubility of ettringite increased as the temperature rised^26^. when the curing temperature increased, the phenomenon of particle agglomeration gradually enhanced. When magnified by 2000 times, the boundary of some particles could be observed. Especially, large agglomerates (particle size of about 10–30 μm) appeared in the sample at 40 °C, and the formation of agglomerates was conducive to improving the strength of solidified silt soil^27^. Figure 8d–f depict representative SEM photographs of the samples cured for 90 days (taking sample F4G6 as an example). When the sample curing temperature is 20 °C, the voids of the sample are obviously smaller than those when the sample is cured for 28 days, which is analyzed macroscopically as an increase in the strength of the sample; when the sample curing temperature is 30 °C, a large number of gel substances such as C–S–H appear, and the increase in the curing temperature accelerates the hydration reaction and pozzolanic reaction between the substances, and the internal voids of the sample become denser; when the sample curing temperature is 40 °C, As shown in Fig. 8f, needle-like ettringite could be observed on the surface of the particles and in the pores, and the voids between soil particles are obviously denser and more compact, and the generation of a large number of gel substances promotes the coupling of filling and cementation between soil particles. The structural features in Fig. 8a–f further confirm the accuracy of the above strength analysis results. According to the analysis results of SEM, C–S–H appeared in all three test samples, and its main microscopic morphology included fibrous, network and foil. Its specific morphological structure was affected by multiple factors, such as curing temperature, curing age and additive addition^28^. Thereinto, the irregular spherical structure is an intermediate of the C–S–H transformation to foil (final state)^29–32^. The hydration product of C–S–H can significasntly improve the strength of dredged silt soil^33^, which is consistent with the influence law of the strength of silt soil in section “UCS”.

### XRD analysis

**Figure 9:**
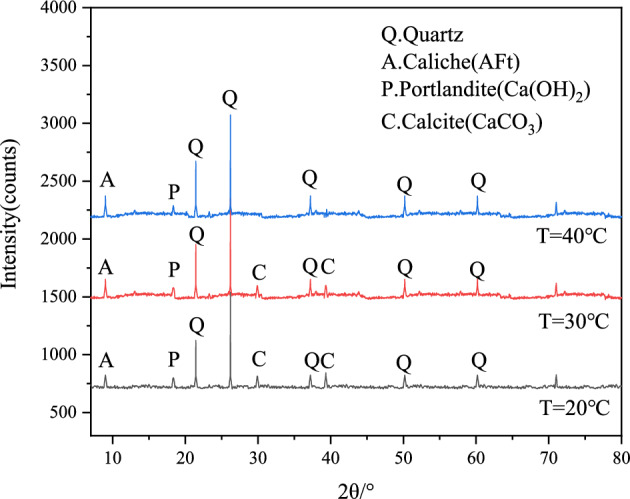
XRD patterns of sample F4G6 at different curing temperatures at 28 days.

**Figure 10:**
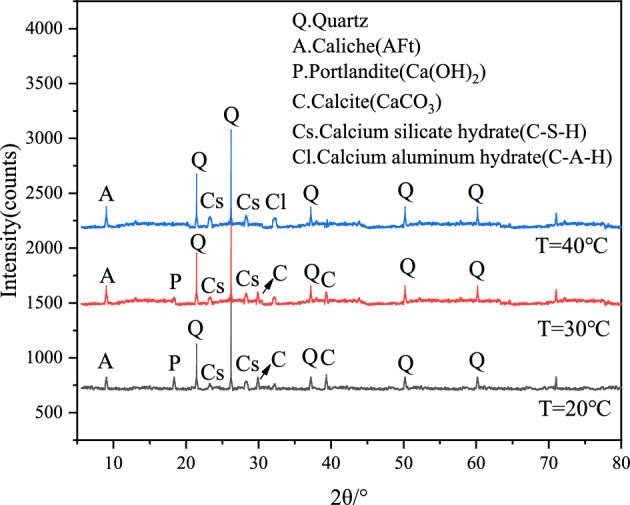
XRD patterns of sample F4G6 at different curing temperatures at 90 days.

After UCS test, the middle small sample was taken and dried at 60 °C, then passed through a 70 μm sieve for backup. XRD (MiniFlex600) of Rigaku Co., Ltd. was used for XRD test, with a scanning speed of 5 (°)/min and a scanning range of 10°-80°. The mineral composition was analyzed by Jade software (link: http://icddchina.com. version: Jade 6.5). Under the curing temperature of 20 °C, 30 °C and 40 °C (taking the sample F4G6 as an example), the representative XRD pattern of the sample cured for 28 and 90 days are shown in Figs. 9 and 10. The C–S–H and C–A–H phases were not detected by XRD when sample F4G6 was cured for 28 days at different curing temperatures, and the C–S–H and C–A–H phases were detected by XRD when sample F4G6 was cured for 90 days at different curing temperatures and appeared as a bulge. Under the precondition of curing at 20 ℃ for 90 days, in addition to the most significant quartz diffraction peak, calcium hydroxide(Ca(OH)_2_) and calcium carbonate(CaCO_3_) phases were detected in F4G6, and the diffraction angle was 18.3° for Ca(OH)_2_ and 29.6° and 39.6° for CaCO_3_. Under the precondition of curing at 30 ℃ for 90 days, a large number of ettringite phases appeared in F4G6, and gel phase substances such as C–S–H were also detected, which accurately confirmed Eq. (2). With the increase of temperature, lime-fly ash-GGBS and other substances interacted with the particles in the silt soil, and the generated gel substances played a role in filling the pores. The sample F4G6 was cured at 40 ℃ for 90 days, and there were more C–S–H and C–A–H phases appeared, exerting a cementing role in the process of solidifying the silt. It is confirmed that under the combined action of the three activators, the increase of the curing temperature promotes the hydration reaction and the pozzolanic reaction, thereby improving the strength of the silt soil, which is consistent with the results of the microscopic test in section “SEM analysis”.

2 $$\begin{gathered} SiO_{2} { + }C{\text{a}}^{2 + } + 2OH^{ - } \mathop{\longrightarrow} CaO \cdot SiO_{2} \cdot H_{2} O(C - S - H) \hfill \\ A{\text{l}}_{2} O_{3} { + }C{\text{a}}^{2 + } + OH^{ - } + SO_{4}^{2 - } \mathop{\longrightarrow}3CaO \cdot A{\text{l}}_{2} O_{3} \cdot 3C{\text{a}}SO_{4} \cdot 32H_{2} O(C - A - S - H) \hfill \\ \end{gathered}$$

## Discussion

This paper focuses on the study of the effect of curing temperature in the cured of slit using lime-activated fly ash-GGBS fly, and analyzes the micro-morphology and mineral composition of the samples using SEM and XRD. The mechanism of the influence of the curing temperature on the lime-fly ash-GGBS triad can be inferred as followings. (1) The study shows that when the curing temperature of the sample is increased from 20 to 40 °C, the hydration reaction and pozzolanic reaction between the raw materials can be accelerated to improve the strength of the sample. In the actual engineering construction process, the effect of the curing temperature on the raw materials can be considered appropriately, so as to reduce the construction cost and improve the utilization rate of solid waste. (2) The use of lime can stimulate the physicochemical reaction between fly ash and GGBS, and substances such as Ca(OH)_2_ and CaCO_3_ can be clearly observed from the XRD patterns, which further indicates that carbonation and hydration reactions have occurred between the three, and substances such as C–S–H and C–A–H can be observed from the SEM and XRD patterns. It is worth noting that the peaks of C–A–H and C–S–H are detected by XRD patterns are low because both are gel phases and non-crystalline structures. The maximum curing temperature studied in this paper is 40 °C. The next step will be to continue to study the mechanism of higher temperature on the effect of curing temperature in the cured of slit using lime-activated fly ash-GGBS, which will provide a valuable reference for the optimal design for dredging silt at high curing temperatures.

## Conclusions

The present paper conducted in-depth study on the effect of curing temperature on the strength and micro-characteristics of lime-activated fly ash-GGBS solidified silt soil. From the three aspects of UCS, SEM and mineral composition analysis of the sample, it is inferred that the strength and microscopic characteristics of the curing temperature on the lime-activated fly ash-GGBS solidified silt soil are mainly reflected in the following four aspects:

1. With the increase of the curing temperature of the sample, the strength of the lime-activated fly ash-GGBS solidified silt soil increased. When the curing temperature increased from 20 to 40 °C, the 28-day strength of F8G2, F6G4, F4G6, F2G8 and F0G10 was 1.05, 1.22, 1.30, 1.36 and 1.43 times that of the original sludge solidified body with 10% cement as curing agent after curing to 28 days. The later strength of lime-activated fly ash-GGBS solidified silt soil was promoted as the curing temperature rises. The hydration reaction and pozzolanic reaction between lime-fly ash-GGBS and silt soil were promoted with the increase of the curing temperature. This conclusion provides a research basis for the resource disposal of river bottom silt and waste solid materials.
2. When the curing temperature increased from 20 to 30 °C, the number of needle-like ettringite increased significantly, and a relatively dense network structure was formed between the particles, and foil-like C–S–H was observed, indicating that the strength of the sample was improved. When the curing temperature reached 40 °C, the morphology of ettringite in the sample changed, mostly in short rod-like form, and the observed ettringite crystals decreased, while the cementing substances such as C–S–H sharply increased.
3. With the increase of curing temperature and curing age, a large number of C–A–H and C–S–H phases were detected by XRD. Such significant particle agglomeration of these cementing substances effectively increased the strength of solidified sludge.
4. Under the experimental conditions in this paper, the curing temperature has achieved a favorable effect on the strength of lime-activated fly ash-GGBS solidified silt soil. When applied to actual engineering, appropriate temperature and insulation measures can be selected in the process of construction and curing, so as to improve the solidification effect of dredged silt and reduce the corresponding cost.

## Data Availability

The data that support the findings of this study are available on request from the corresponding authors.
